# Fornix-Based versus Limbus-Based Conjunctival Flap in Trabeculectomy: A Quantitative Evaluation of the Evidence

**DOI:** 10.1371/journal.pone.0083656

**Published:** 2013-12-26

**Authors:** Wei Wang, Miao He, Minwen Zhou, Xiulan Zhang

**Affiliations:** Zhongshan Ophthalmic Center, State Key Laboratory of Ophthalmology, Sun Yat-Sen University, Guangzhou, People's Republic of China; Massachusetts Eye & Ear Infirmary, Harvard Medical School, United States of America

## Abstract

**Objective:**

To evaluate the efficacy and tolerability of limbus-based (LBCF) compared with fornix-based conjunctival flaps (FBCF) for trabeculectomy in the treatment of patients with uncontrolled glaucoma.

**Methods:**

A comprehensive literature meta-analysis was performed according to the Cochrane Collaboration methodology to identify controlled clinical trials comparing LBCF with FBCF in trabeculectomy. The efficacy measures were the weighted mean differences (WMDs) for intraocular pressure reduction (IOPR), the reduction in glaucoma medications, and the relative risks (RRs) for success rates. Tolerability estimates were measured by RR for adverse events. The pooled effects were calculated using the random effects model.

**Results:**

Sixteen controlled clinical trials meeting the predefined criteria were included in the meta-analysis. A total of 1,825 eyes from 1,392 patients with medically uncontrolled glaucoma were included. The WMD of the IOPR from baseline was 1.12 (95% CI: −0.88 to 3.12) when comparing LBCF with FBCF. LBCF was associated with numerically greater but non-significant IOP lowering efficacy than FBCF (P = 0.270). LBCF was comparable with FBCF in the reduction of glaucoma medication, with a WMD of 0.15 (−0.05 to 0.36) at the follow-up endpoint (P = 0.141). The pooled RR comparing LBCF with FBCF were 1.08 (0.94, 1.23) for the complete success rate and 1.01 (0.92, 1.10) for the qualified success rate. Rates of adverse events did not differ significantly between LBCF and FBCF.

**Conclusions:**

There is no significant difference in IOP lowering, number of glaucoma medications, or proportion of patients who reached the IOP target between LBCF and FBCF trabeculectomy. Both incision techniques may contribute equally to adverse events.

## Introduction

Glaucoma is the second leading cause of blindness worldwide and constitutes a major global healthcare problem [1]. It has been estimated that, in 2010, there were approximately 60 million glaucoma patients worldwide, and this figure is expected to rise to 80 million by 2020 [2]. Since it was introduced in 1968 by Cairns, trabeculectomy has remained the most common surgical procedure for treatment of glaucoma [3]. In the past, several approaches to conjunctival dissection during trabeculectomy have been described. Incisions through the conjunctiva made posterior to the limbus are called limbus-based conjunctival flaps (LBCF), and those where the conjunctiva is incised at the limbus are called fornix-based conjunctival flaps (FBCF) [3].

Several clinical trials have compared the efficacy and safety of LBCF versus FBCF. However, of the four prospective randomized studies and two retrospective studies, four found no difference between the two conjunctival approaches, and two found superior IOP lowering with the FB approach [4]–[9]. Two of the studies reported a higher likelihood of early post-operative aqueous leaks with FB incisions [10], [11]. Thus, evidence is mixed as to which conjunctival incision is superior for trabeculectomy. In 2005, a narrative review was designed to determine differences between the two techniques in the resultant intraocular pressure control and complication rate. Based the insufficient information, they concluded that a FBCF trabeculectomy may be more favorable than the LBCF approach [12]. These seemed not solid since they were based on a descriptive analysis of relevant small trials, not a meta-analysis, a statistical method with high confidence in achieving scientific results. Thus, it is necessary to compare the efficacy and safety of LBCF versus FBCF with a well-conducted evidence-based analysis. Therefore, the present meta-analysis of controlled comparative trials, comparing LBCF and FBCF, was undertaken to assess the efficacy and tolerability of both techniques for trabeculectomy in the treatment of uncontrolled glaucoma.

## Methods

This meta-analysis was performed according to a predetermined protocol described in the following paragraph using standard systematic review techniques, as outlined by the Cochrane Handbook for Systematic Reviews of Interventions and Preferred Items for Systematic Reviews and Meta-Analysis (PRISMA) Statement (Table S1) [13].

### 1. Literature search

Five electronic databases–PubMed, ISI Web of Science, EMBASE, the Chinese Biomedicine Database, and the Cochrane Library–were searched systematically for studies published before August 2013. The following terms, adapted for each database, were used for the searches: limbus-based, fornix-based, conjunctival flap, trabeculectomy, and glaucoma. The Internet was searched using the Google search engine. A manual search was performed by checking the reference lists of original reports and review articles, retrieved through electronic searches, to identify studies not yet included in the computerized databases. The final search was carried out on 1 August 2013, without restrictions regarding publication year, language, or methodological filter.

### 2. Inclusion and exclusion criteria

The articles were considered eligible if the studies met the following inclusion criteria: (i) study type: comparative studies; (ii) population: adult (≥18 years old) glaucoma patients for whom conservative therapy fail; (iii) intervention: LBCF versus FBCF trabeculectomy, with or without the use of antimetabolites and with or without concurrent cataract surgery; (iv) at least one of the outcome measures was required, and the follow-up time was no less than six months. Abstracts from conferences and full texts without raw data available for retrieval, duplicate publications, letters, and reviews were excluded. For publications reporting on the same study population, the article reporting the results of the last end point was included, and data that could not be obtained from this publication were obtained from others.

### 3. Outcome measures

For efficacy, the primary outcome was IOP reduction (IOPR). When authors reported the mean and standard deviation (SD) of the IOPR, we used these directly. For studies that only reported absolute values for the IOP at baseline and the end point, the IOP reduction (IOPR) and the SD of the IOPR (SD_IOPR_) were calculated as follows: IOPR  =  IOP_baseline_ − IOP_end-point_, SD_IOPR_  =  (SD_baseline_^2^ + SD_end-point_^2^ − SD_baseline_ × SD_end-point_)^1/2^ [14]. The secondary outcome measure was the difference in reduction with glaucoma medications. For efficacy, the proportion of complete success and qualified success were also used. Complete success was defined as the target end-point IOP without medications, and qualified success was defined as the target end-point IOP with or without medications. We assessed tolerability by considering the proportions of patients with postoperative complications in either group.

### 4. Data extraction

The data were extracted independently by two reviewers (W.W. and M.H.) and were rechecked after the first extraction. Disagreements were resolved through discussion. The information extracted from each study included the authors of each study, the year of publication, information on the study design, the location of the trial, the duration of the study, the number of subjects, age, sex, IOP measurements, the number of glaucoma medications, and success rate. The numbers of withdrawals and patients reporting adverse events were also recorded.

### 5. Assessment of methodology quality

The qualities of the included clinical trials were assessed by two independent observers (W.W. and Z.M.W.) using a system that can assess both randomized and non-randomized studies. This system was previously reported on by Downs and Blacks [15]. The system comprises 27 items distributed among five subscales regarding reporting (10 items), external validity (three items), bias (seven items), confounding (six items), and power (one item). Any discrepancy in the qualitative assessment between the two observers was discussed, and a consensus was reached. The total score for each trial was expressed as a percentage of the maximum achievable score. The studies with a quality score ≥50% were considered to have adequate quality.

### 6. Statistical analysis

The outcome measures were assessed on an ITT basis. Given that some of the trials did not report all the outcomes of interest, for each comparison and outcome, we conducted separate meta-analyses. Considering the differences in the clinical characteristics among study groups and the variations in sample size, it was assumed that heterogeneity was present even when no statistical significance was identified, and it was decided to combine data using a random-effects model [16]. A pooled risk ratio (RR) with a 95% confidence interval (CI) was calculated for dichotomous outcomes. For the continuous outcomes, the weighted mean difference (WMD) with a 95% CI was calculated. Statistical heterogeneity among studies was evaluated with the χ2 and I^2^ tests [17]. P<0.05 was considered statistically significant for the test for overall effect. All statistical analyses were performed using Stata (version 12; StataCorp, College Station, Texas).

### 7. Sensitivity analysis and publication bias

Sensitivity analysis was undertaken to evaluate the effect of the methodological characteristics of controlled clinical trials in terms of trial design, which was differentiated as retrospective, prospective non-randomized, and randomized. Furthermore, a subgroup analysis was carried out to evaluate the impact of the surgical characteristics on the results. To detect publication biases, we explored asymmetry in funnel plots. These were examined visually; furthermore, the Begg and Egger measures were calculated [18], [19].

## Results

### 1. Literature search

**Figure 1:**
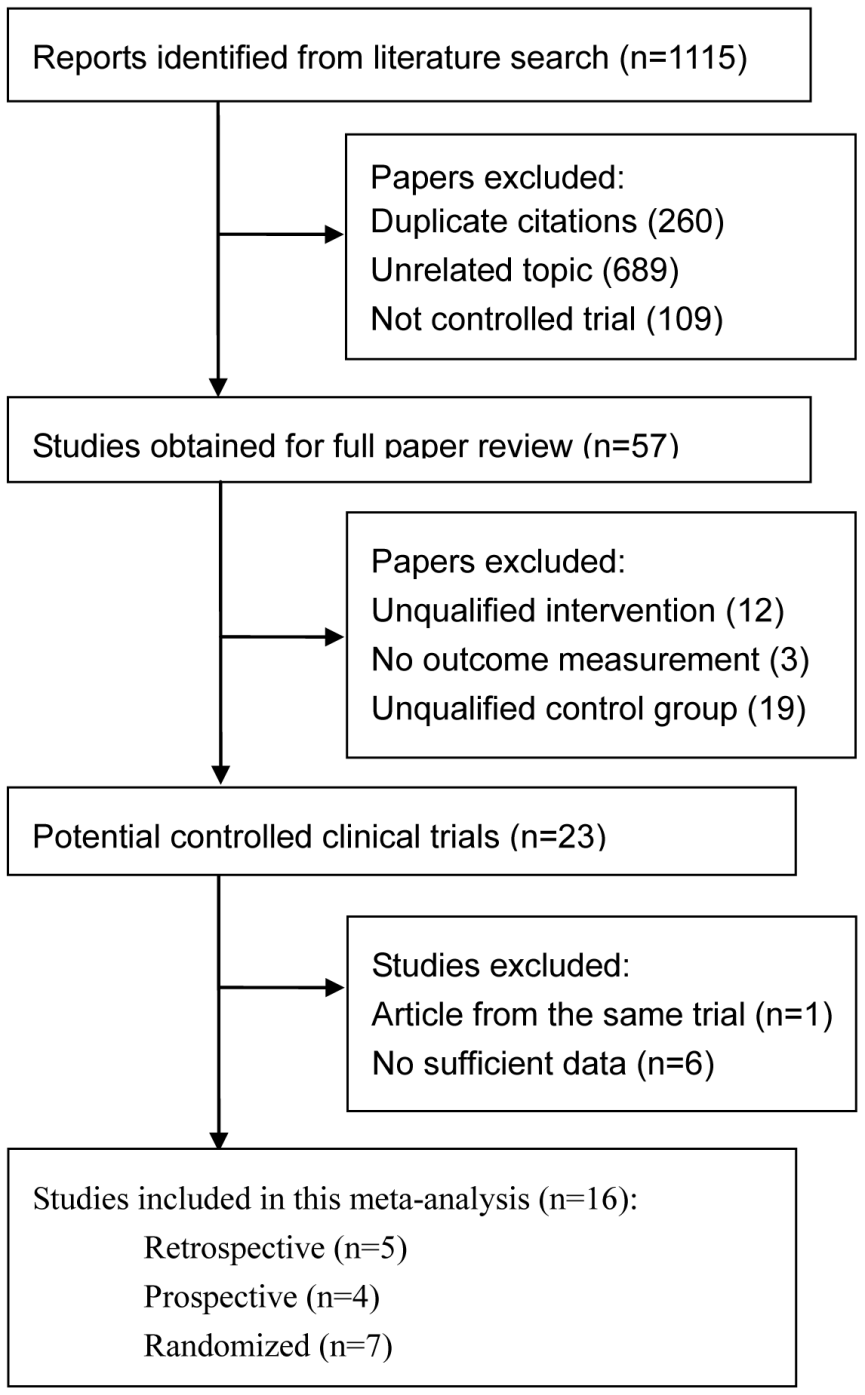
Flow diagram of included studies for this meta-analysis.

A total of 1,115 articles were initially identified. The abstracts were reviewed, and 57 articles with potentially relevant trials were reviewed in their entirety. Subsequently, 23 articles with full texts that met the inclusion criteria were assessed[4]–[11], [20]–[34]. Seven studies were excluded: six lacking primary outcome data and two reporting on the same subjects integrated into one study. Thus, a total of 16 studies were included in the final meta-analysis[4]–[11], [21], [24], [25], [27], [29], [31]–[33]. Figure 1 provides a flow diagram of the search results.

### 2. Characteristics and quality of the included studies

In total, 1,825 eyes from 1,392 patients were included in this meta-analysis; 959 were assigned to the LBCF group and 866 to the FBCF group. The characteristics of the studies included are summarized in Table 1. The percentage of included males ranged from 19.4% to 69%, and the mean age of the study patients ranged from 51.3 to 78.6 years. The study design was retrospective in five studies, prospective non-randomized in four studies, and randomized clinical trials (RCT) in seven studies. The studies were conducted in U.S.A., Denmark, Germany, Saudi Arabia, Greece, China, and other countries. The mean duration of follow-up ranged from 6 to 48 months. Eleven of the 16 studies used antimetabolites intraoperatively or postoperatively; among them, 10 reported using mitomycin C with concentrations varying from 0.05 to 0.4 mg/ml for a duration of 1 to 5 minutes. The methodological quality of the studies was average. Not all studies clearly defined their outcome measures, thereby increasing heterogeneity, and none included a blinded assessment of the endpoints, which could have led to expectation bias.

**Table 1:** Characteristics of studies in the meta-analysis.

First author (year)	Country	Design	Eyes*	Patients	Follow-up(m)	Age(y)	Male(%)	Combine surgery	Use of antimetabolite
Shuster(1984)	USA	RCT	18/19	18/19	17.6/16.0	65.4/62.8	NA	no	no
Brincker(1992)	Denmark	RCT	18/18	18	6/6	70.2	44.4%	no	MMC
Auw-Haedrich(1998)	Germany	RCT	43/47	81	NA	73.6/78.6	NA	no	no
Lemon(1998)	USA	RCT	30/39	NA	14.1/15.9	73.6/74.9	40%	+phaco	MMC
Sayyad F(1999)	Saudi Arabia	RCT	29/29	29	48/48	51.3	69.0%	no	5-Fu
Kozobolis(2002)	Greece	RCT	30/30	30	12/12	71.4	60.0%	+phaco	MMC
Cheng(2012)	China	RCT	72/76	64/68	12/12	59.4	50.0%	no	no
Khan(1992)	India	Pro	50/50	50/50	12/12	NA	46.0%	no	no
Stewart(1994)	USA	Pro	15/16	15/16	NA	76.2/71.8	19.4%	+phaco	no
Shingleton(1999)	USA	Pro	44/44	44	12/12	75.4	47.7%	+phaco	MMC
Mandic(2004)	Croatia	Pro	16/16	16	20	65.0	37.5%	+phaco	MMC
Berestka(1997)	USA	Retro	28/24	24/21	26/12	75.6/76.3	50.3%	+phaco	MMC
Alwitry(2005)	UK	Retro	35/36	27/32	6	69.7/69.8	30.5%	no	MMC
Fukuchi(2006)	Japan	Retro	44/38	44/38	20.7/20.2	60.5/62.8	46.3%	no	MMC
Lin(2007)	USA	Retro	42/32	31/23	12/12	62.7/62.6	53.7%	no	MMC
Solus(2012)	USA	Retro	445/352	634	48	65.6	43.5%	no	MMC+5-Fu

Limbus-based conjunctival flaps group/Fornix-based conjunctival flaps group; m: months; y: years; RCT: prospective randomized controlled trial; Retro: retrospective comparative study; Pro: prospective non-randomized comparative study; NA: not available; phaco: phacomucificaion; MMC: mitomycin C; 5-FU, 5-fluorouracil.

### 3. Efficacy analysis

**Figure 2:**
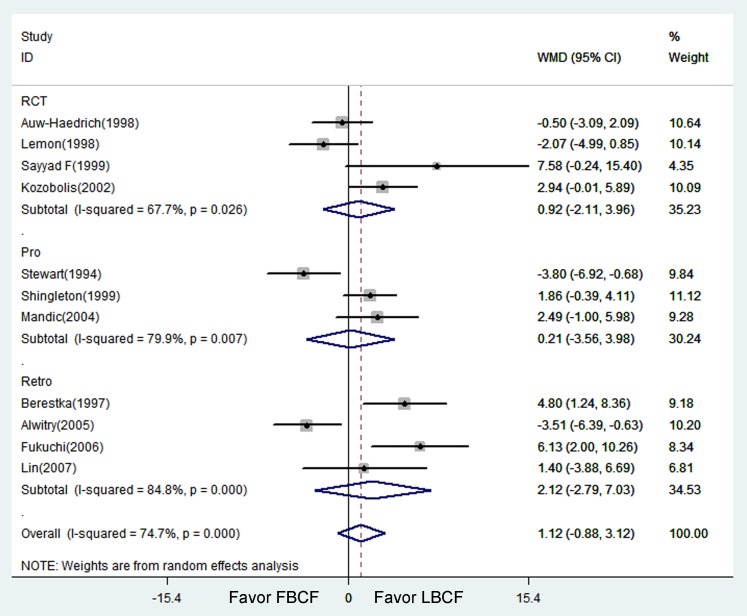
Forest figure of IOPR comparing Limbus- with Fornix-based trabeculectomy. (Weighted mean differences (WMD) were computed by using a random effects model. 95% CI indicates 95% confidence interval; RCT: prospective randomized controlled trial; Retro: retrospective comparative study; Pro: prospective non-randomized comparative study.)

Eleven studies involving 744 eyes compared LBCF with FBCF in terms of the IOPR. Substantial statistical heterogeneity was observed between studies (χ2 = 39.49, P<0.001, I^2^ = 75%). LBCF was found to achieve a numerically greater IOPR from baseline; however, the differences in the IOPR were not statistically significant (WMD = 1.12, 95% CI: −0.88 to 3.12; P = 0.270) (Table 2). We then divided the studies into three subgroups according to the study design (retrospective, prospective non-randomized, and randomized) (Figure 2). All the subgroups showed that LBCF was associated with a numerically higher IOPR relative to FBCF, but no significant difference was found. Similar primary outcomes were obtained when the analysis was stratified by trabeculectomy and phocotrabeculectomy. There was substantial heterogeneity in these analyses. For the subgroup analysis according to the use of antimetabolites, the difference between groups was not statistically significant, and no statistical heterogeneity was shown between studies (χ2 = 2.55, P = 0.61, I^2^ = 11% without the use of antimetabolite; χ2 = 29.12, P = 0.73, I^2^ = 0% with the use of antimetabolite) (Table 2).

**Table 2:** Intraocular pressure reduction (IOPR) from baseline comparing Limbus- with Fornix-based trabeculectomy.

		NO. of studies	WMD (95% CI)			Test for Heterogeneity			Test for Overall Effect	
			Estimate	Lower	Up	?2	I^2^	P	Z	P
All		11	1.12	−0.88	3.12	39.49	75%	<0.001	1.09	0.270
Design										
	RCT	4	0.92	−2.11	3.96	9.3	68%	0.03	0.6	0.550
	Pro	3	0.21	−3.56	3.98	9.93	80%	0.01	0.11	0.910
	Retro	4	2.12	−2.79	7.03	19.69	85%	<0.001	0.85	0.400
Combine surgery										
	No	5	1.54	−2.27	5.34	18.20	78%	0.001	0.79	0.428
	Yes	6	0.99	−1.49	3.47	20.61	75.7%	0.001	0.78	0.435
Use of antimetabolite										
	No	2	−2.03	−5.25	1.20	2.55	11%	0.61	1.23	0.218
	Yes	9	1.94	−0.29	4.16	29.12	00%	0.73	1.71	0.088

Weighted mean differences (WMD) were computed by using a random effects model. 95% CI indicates 95% confidence interval; Limbus: Limbus-based conjunctival flaps group; Fornix: Fornix-based conjunctival flaps group; RCT: prospective randomized controlled trial; Retro: retrospective comparative study; Pro: prospective non-randomized comparative study.

With respect to glaucoma medication reduction, the pooled WMD for LBCF was found to be 0.15 compared with FBCF, the difference was not significant with a 95% CI, which was −0.05 to 0.36 (P = 0.141). No contradictory significant differences were observed in the results of the sensitivity analysis compared to the previous analysis. No significant heterogeneity between study results was detected in these analyses (Table 3).

**Table 3:** The reduction in glaucoma medication from baseline comparing Limbus- with Fornix-based trabeculectomy.

		NO. of studies	WMD (95% CI)			Test for Heterogeneity			Test for Overall Effect	
			Estimate	Lower	Up	?2	I^2^	P	Z	P
All		5	0.15	−0.05	0.36	0.59	<0.001	0.965	1.47	0.141
Design										
	RCT	2	0.14	−0.18	0.45	0.53	<0.001	0.466	0.86	0.391
	Pro	2	0.17	−0.14	0.48	0.04	<0.001	0.849	1.07	0.286
	Retro	1	0.16	−0.40	0.72	-	-	-	0.56	0.576
Combine surgery										
	no	1	0.16	−0.40	0.72	-	-	-	0.56	0.576
	yes	4	0.15	−0.07	0.37	0.59	<0.001	0.9	1.36	0.173

Weighted mean differences (WMD) were computed by using a random effects model. 95% CI indicates 95% confidence interval; Limbus: Limbus-based conjunctival flaps group; Fornix: Fornix-based conjunctival flaps group; RCT: prospective randomized controlled trial; Retro: retrospective comparative study; Pro: prospective non-randomized comparative study.

**Figure 3:**
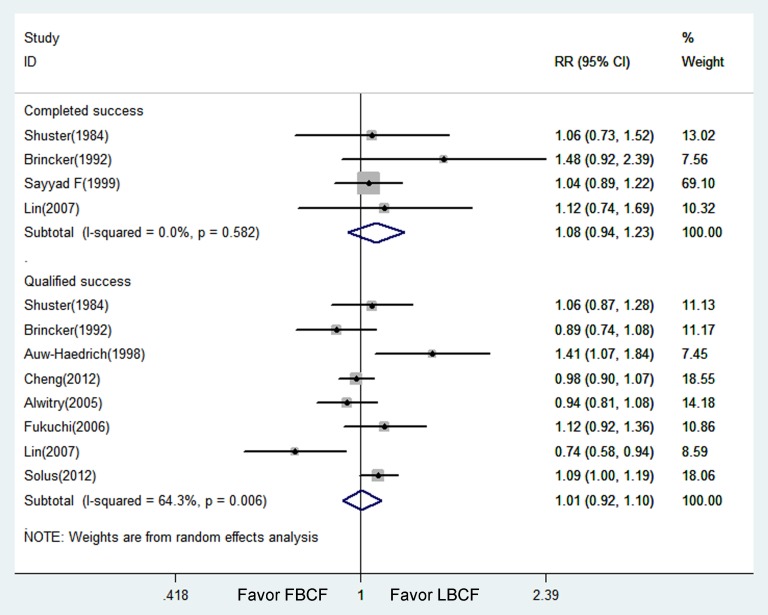
Forest figure of success rate comparing Limbus- with Fornix-based trabeculectomy. (Relative risks (RR) were computed by using a random effects model. 95% CI indicates 95% confidence interval; RCT: prospective randomized controlled trial; Retro: retrospective comparative study; Pro: prospective non-randomized comparative study.)

Concerning the success rate, four studies reported the probability of complete success, and no significant difference was found between LBCF and FBCF (pooled RR = 1.08, 95% CI: 0.94–1.23; P = 0.267) (Table 4) (Figure 3). There was also no significant difference in complete success between LBCF and FBCF in the sensitivity analyses according to study design and use of antimetabolite. Eight studies reported the proportion of patients achieving target end-point IOP with or without medications at the follow-up end point; the difference in the qualified success rate between the LBCF group and the FBCF group was not statistically significant (pooled RR = 1.01, 95% CI: 0.92–1.10; P = 0.864) (Figure 3). For the subgroup analysis, no statistical heterogeneity was shown between studies, and the difference between groups was not statistically significant (Table 4).

**Table 4:** Complete success and qualified success rates comparing Limbus- with Fornix-based trabeculectomy.

	NO.of studies	Success Rate, n/N (%)		RR (95% CI)			Test for Heterogeneity			Test for Overall Effect	
		Limbus	Fornix	Estimate	Lower	Up	?2	I^2^	P	Z	P
Completed success rate											
All	4	81/107	66/96	1.08	0.94	1.23	1.95	0.00%	0.582	1.11	0.267
RCT	3	56/65	49/64	1.07	0.93	1.23	1.91	0.00%	0.384	0.99	0.323
Retro	1	25/42	17/32	1.12	0.74	1.69	-	-	-	0.54	0.587
no-MMC	1	14/18	14/19	1.06	0.73	1.52	-	-	-	0.29	0.772
MMC	3	67/89	52/77	1.08	0.94	1.25	1.94	0.00%	0.379	1.08	0.281
Qualified success rate											
All	8	495/606	456/584	1.01	0.92	1.10	19.61	64.30%	0.006	0.17	0.864
RCT	4	136/151	135/160	1.04	0.90	1.19	7.99	62.40%	0.046	0.49	0.626
Retro	4	359/455	321/424	0.98	0.84	1.14	11.40	73.70%	0.010	0.28	0.779
no-MMC	3	120/133	117/142	1.09	0.91	1.31	6.34	68.50%	0.042	0.97	0.332
MMC	5	375/473	339/442	0.96	0.85	1.10	13.23	69.80%	0.010	0.56	0.575

RR indicates relative risk, which was computed by using a random effects model. 95% CI indicates 95% confidence interval; n, number of patients achieving target endpoint intraocular pressure; N, number of patients; Limbus: Limbus-based conjunctival flaps group; Fornix: Fornix-based conjunctival flaps group; RCT: prospective randomized controlled trial; Retro: retrospective comparative study; Pro: prospective non-randomized comparative study; MMC, mitomycin C.

### 4. Tolerability analysis

Adverse events in controlled clinical trials comparing LBCF and FBCF are shown in Table 5. Bleb leak and a flat anterior chamber were two of the most commonly reported postoperative adverse events. No significant differences between LBCF and FBCF were found with respect to the incidence of bleb leak, flat anterior chamber, hyphema, choroidal effusion, early postoperative hypotony, bleb fibrosis, hypotony maculopathy, needling, endophthalmitis, suprachoroidal hemorrhage, and bleb revision.

**Table 5:** Adverse events from Limbus- and Fornix-based trabeculectomy compared.

Adverse event	NO.of studies	Crude Rate, n/N (%)		RR (95% CI)			Test for Heterogeneity			Overall Effect	
		Limbus	Fornix	Estimate	Lower	Up	?2	I^2^	P	Z	P
Bleb leak	12	58/697	83/671	0.70	0.35	1.42	29.14	62.30%	0.002	0.99	0.321
Flat anterior chambe	11	63/397	41/390	1.38	0.97	1.98	7.70	0.00%	0.658	1.78	0.076
Hyphema	9	28/311	28/309	0.95	0.59	1.53	3.60	0.00%	0.892	0.21	0.834
Choroidal effusion	7	35/232	30/213	1.04	0.66	1.62	4.04	0.00%	0.671	0.16	0.876
Early postoperative hypotony	6	85/533	57/525	1.47	0.95	2.28	6.57	23.90%	0.254	1.71	0.087
Bleb fibrosis	3	10/76	14/76	0.72	0.35	1.49	0.02	0.00%	0.988	0.89	0.374
Hypotony maculopathy	3	2/103	2/106	1.02	0.18	5.76	0.75	0.00%	0.688	0.02	0.983
Needling	3	9/93	16/99	0.52	0.25	1.08	0.66	0.00%	0.718	1.76	0.079
Endophthalmitis	2	2/369	4/354	0.66	0.06	7.64	1.70	41.20%	0.192	0.34	0.736
Suprachoroidal hemorrhage	1	1/15	0/16	3.19	0.14	72.689	-	-	-	0.73	0.467
Bleb revision	1	1/42	1/32	0.76	0.05	11.722	-	-	-	0.19	0.845

RR indicates relative risk, which was computed by using a random effects model. 95% CI indicates 95% confidence interval; n, number of patients with adverse events; N, number of patients; Limbus: Limbus-based conjunctival flaps group; Fornix: Fornix-based conjunctival flaps group.

### 5. Publication bias

Publication bias for the primary outcome was tested using Begg's test (P =  0.161) and Egger's test (P =  0.233), and no obvious evidence of publication bias was found.

## Discussion

A persistent source of controversy in trabeculectomy has been the orientation of the conjunctival flap[3]. With data from 16 clinical controlled studies, this meta-analysis summarizes the available evidence on outcomes of trabeculectomy with LBCF or FBCF. The pooled results showed that both LBCF and FBCF were safe and effective for trabeculectomy. There was no significant difference in IOP lowering, glaucoma medication reduction, success rate and adverse event rates. Furthermore, the results from the subgroup and sensitivity analyses by research design, use of antimetabolite, and combination of phacomulcification were quite similar and robust.

Intraoperative mitomycin C (MMC) and postoperative use of 5-fluorouracil (5-FU) injections are beneficial to the eyes at a high risk of failure[35]. Trabeculectomy can also be performed concomitantly with phacoemulsification[36]. Care must be taken when attempting to extrapolate data from studies addressing combined cataract and trabeculectomy procedures compared to trabeculectomy procedures alone. The stimulus for wound healing is thought to be very different in eyes subjected to combined procedures[3]. A number of investigators have advocated the use of FBCF instead of LBCF[12]. Theoretically, FBCF with no incision through Tenon's capsule may offer an untouched area for aqueous humor resorption, while LBCF may result in a dense scar at the site where the conjunctiva and Tenon's capsule are cut[29], [30]. However, the present study detected no difference between eyes with FBCF and those with LBCF in the final outcomes. When an antimetabolite augmented trabeculectomy or was combined with phacomulcificaion, the results were similar.

Complications as a result of trabeculectomy remain a troubling side effect for surgeons and patients. Twelve studies reported complications related to the two techniques. The main complications in these studies were bleb leak and a flat anterior chamber. Because the commonly used LBCF method can make tighter conjunctival sutures, there is less chance of postoperative leakage. Moreover, there was a tendency for the increased occurrence of a shallow anterior chamber in the LBCF group (OR = 1.38, 95% CI: 0.97 to 1.98; P = 0.076). This may be due to the increased exposition of sclera and subconjunctival tissue with this technique: the initial outflow resistance in the subconjunctival space may be lower, as the initial hypotony was more pronounced when the subconjunctival tissue was opened over a larger area[37], [38]. However, no significant between-group differences in complications were found in the current meta-analysis.

Theoretically, LBCF makes a conjunctival suture line that adheres to the sclera. Long-term scarring and shrinkage of this line may result in weakening of the bleb wall and may contribute to bleb-related infection[34], [39]. The results of the meta-analysis of the incidence of endophthalmitis, which was based on only two studies, indicated no difference between the two techniques. It could not be confirmed whether or not LBCF was associated with a greater risk of infection based on so few studies. More studies would be warranted.

Between-study heterogeneity in the present meta-analysis was not significant for most dichotomous outcomes but was significant for most of the continuous variables. The included studies adopted various matching criteria, operative techniques. In addition, the variability in the surgeon experience, different surgical indications, and non-standardized measurement of outcomes may have introduced potential bias. The pooling of data using the random-effects model might reduce the effect of heterogeneity but does not abolish it.

The clinical relevance of these results must be interpreted with caution. The present study has limitations that stem from the designs of the individual trials, as well as the methods of the meta-analysis itself. In an attempt to review the literature, we were surprised to discover only a very few randomized studies evaluating the efficacy and tolerability of LBCF compared with FBCF for trabeculectomy. Thus, the main limitation of this review is the small number of randomized controlled trials. A second limitation is that our analyses were based on data pooled from trials of different durations due to the lack of data reported in all phases of follow-up. It was a compromise proposal to choose the data from the follow-up endpoint. A third potential limitation is that the criteria for success differed among studies. Some used only IOP measurement, while others also looked into visual field progression, optic disc cupping, and loss of visual acuity[4], [11], [22], [24], [40]. Although such assessments of success are widely used as outcome measures in clinical trials, further research is still needed to determine fully their validity, reliability, and sensitivity to choose the best one. A fourth limitation of this meta-analysis is that the follow-up time was not long enough. Even though longer-term results are lacking, the study by Sayyad et al.[6] with a mean follow-up period of 48 months, indicating the similar efficacy of LBCF and FBCF in trabeculectomy; however, the rates of late-onset bleb leakage were greater in patients with LBCF. With the increase in availability of more studies with longer follow-up with time, there could potentially be a change in the findings of tolerability in this meta-analysis. The fifth limitation of this meta-analysis is that our analysis did not evaluate some important outcomes, such as the morphological features of filtering blebs, surgical time, visual acuity, and the visual field. Shedding more light on the areas mentioned above would ultimately allow a better understanding of the role and value of the two techniques in glaucoma therapy.

Even though our meta-analysis has several limitations, it represents the first systematic attempt to critically appraise the evidence surrounding the difference comparing LBCF and FBCF in trabeculectomy for glaucoma. Multiple strategies were applied to identify the most valid studies currently published. The strict criteria were used to include and evaluate the quality of the studies. The PRISM guidelines were used for the reporting of our systematic review, and non-English studies were included to minimize publication bias. Furthermore, subgroup and sensitivity analyses all confirmed the reliability of the pooled estimates in the meta-analysis. This analysis, therefore, provides the most up-to-date information in this area. In the future, more inclusive and well-designed RCTs are needed to confirm our conclusion.

The present meta-analysis suggests that the two surgical techniques appear to be equivalent in terms of IOP lowering, reduction in the number of glaucoma medications, the success rate, and the rate of adverse events. Nevertheless, despite our rigorous methodology, the inherent limitations of the included studies should be considered, and conclusions drawn from our pooled results should be interpreted with caution. Future large-volume, well-designed RCTs with extensive follow-up are awaited to confirm and update the findings of this analysis.

## Supporting Information

Table S1 **PRISMA checklist.**
(DOC)
